# Supplementary material for: The Ralstonia solanacearum Type III Effector RipAW Targets the Immune Receptor Complex to Suppress PAMP-Triggered Immunity
Source: Int J Mol Sci. 2023 Dec 22;25(1):183. doi: 10.3390/ijms25010183 (PMC10779406; doi:10.3390/ijms25010183)
Supplement: Supplementary file 1 [file ijms-25-00183-s001.zip › ijms-2728497-supplementary.pdf]

Table S1. The primers used in this study

Primer_name	Primer_sequence
1300-ATFLS2-FLAG-F	ACGGGGGACGAGCTCGGTACCATGAAGTTACTCTCAA
1300-ATFLS2-FLAG-R	ATGGTCTTTGTAGTCGACAACTTCTCGATCCTCGTTACG
1300-ATXLG2-FLAG-F	ACGGGGGACGAGCTCGGTACCATGGCTGCAGTTATAA
1300-ATXLG2-FLAG-R	ATGGTCTTTGTAGTCGACAGAGGACGAGCTGGCCTCTA
1300-ATBIK1-FLAG-F	ACGGGGGACGAGCTCGGTACCATGGGTTCTTGCTTCA
1300-ATBIK1-FLAG-R	ATGGTCTTTGTAGTCGACCACAAGGTGCCTGCCAAAA
BIFC-RipAW-F	TTACGAACGATAGTTAATTAATATGGTTTTCCTTGTCCG
BIFC-RipAW-R	CCTCCTCCACTAGTGGCGCGCCCTCCGCCGCGCGCGG
BIFC-BIK1-F	TTACGAACGATAGTTAATTAATATGGGTTCTTGCTTCAG
BIFC-BIK1-R	CCTCCTCCACTAGTGGCGCGCCCCACAAGGTGCCTGC
BIFC-FLS2-F	TTACGAACGATAGTTAATTAATATGAAGTTACTCTCAA
BIFC-FLS2-R	CCTCCTCCACTAGTGGCGCGCCCAACTTCTCGATCCTC
BIFC-XLG2-F	TTACGAACGATAGTTAATTAATATGGCTGCAGTTATAAG
BIFC-XLG2-R	CCTCCTCCACTAGTGGCGCGCCCAGAGGACGAGCTGG
FRK1-qPCR-F	CAAGCTTTGCTCTTCTGTTCTT
FRK1-qPCR-R	AACCACTTGATTGAAACTGAGC
WRKY29-qPCR-F	TTAGATGAGCTCGGTGAACTTT
WRKY29-qPCR-R	CCATGTGATCGTTGTTTCTTGT
ACT1-qPCR-F	TCTTGATCTTGCTGGTCGTG
ACT1-qPCR-R	GAGCTGGTTTTGGCTGTCTC
RipAW-EcoRI-F	GCCCCCTTCACAGTGAATTCATGGTTTTCCTTGTCCGG
RipAW-HindIII-w/o-R	GACCATGATTACGCCAAGCTTCTCCGCCGCGCGCGGC
RipAW-C177A-F	GTGCCGATGCCGACGCCTATGACAACGCCG
RipAW-C177A-R	CGGCGTTGTCATAGGCGTCGGCATCGGCAC
RipAW-F	ATGACAACGCCGAGGTCATT
RipAW-R	CCCCCTGATTGACACTGGTC
P2300-RipAW-F	AGCTTTCGCGAGCTCGGTACCATGGTTTTCCTTGTCCG
P2300-RipAW-R-FLAG	CTCGAGCTTGCATGCCTGCAGTCACTTGTCATCGTCGT
RipAW- Nluc-F	ACGGGGGACGAGCTCGGTACCATGGTTTTCCTTGTCCG
RipAW- Nluc-R	AACATCGTATGGGTAGTCGACTCCGCCGCGCGCGGCG
